# The oligodendroglial precursor cell line Oli-neu represents a cell culture system to examine functional expression of the mouse gap junction gene connexin29 (Cx29)

**DOI:** 10.3389/fphar.2013.00083

**Published:** 2013-06-28

**Authors:** Goran Söhl, Sonja Hombach, Joachim Degen, Benjamin Odermatt

**Affiliations:** Abteilung Molekulargenetik, Institut für Genetik, Universität BonnBonn, Germany

**Keywords:** gap junction, connexin, Cx29, oligodendroglial precursor cell-line Oli-neu, HeLa cells, dye transfer

## Abstract

The potential gap junction forming mouse connexin29 (Cx29) protein is concomitantly expressed with connexin32 (Cx32) in peripheral myelin forming Schwann cells and together with both Cx32 and connexin47 (Cx47) in oligodendrocytes of the CNS. To study the genomic structure and functional expression of Cx29, either primary cells or cell culture systems might be selected, from which the latter are easier to cultivate. Both structure and expression of Cx29 is still not fully understood. In the mouse sciatic nerve, brain and the oligodendroglial precursor cell line Oli-neu the Cx29 gene is processed in two transcript isoforms both harboring a unique reading frame. In contrast to Cx32 and Cx47, only Cx29 protein is abundantly expressed in undifferentiated as well as differentiated Oli-neu cells but the absence of Etbr dye transfer after microinjection concealed the function of Cx29-mediated gap junction communication between those cells. Although HeLa cells stably transfected with Cx29 or Cx29-eGFP neither demonstrated any permeability for Lucifer yellow nor for neurobiotin, blocking of Etbr uptake from the media by gap junction blockers does suppose a role of Cx29 in hemi-channel function. Thus, we conclude that, due to its high abundance of Cx29 expression and its reproducible culture conditions, the oligodendroglial precursor cell line Oli-neu might constitute an appropriate cell culture system to study molecular mechanisms or putative extracellular stimuli to functionally open Cx29 channels or hemi-channels.

## Introduction

Oligodendrocytes do myelinate neuronal axons in the CNS to allow fast nerve conduction as well as guidance and care for neuronal networks (cf. Kandel et al., 2000). Oligodendroglial progenitor cells (O-2A) can be divided in two different lineages developing from neuro-epithelial precursors in the wall of the embryonic neural tube around mouse embryonic day E12 (Richardson et al., 2000). In the CNS, O-2A cells exist in two subpopulations with different maturation profiles: O-2A^perinatal^ cells are up-regulated in the rat postnatally, providing myelination during this period, but disappear about 6 weeks after birth (Wolswijk and Noble, 1989); O-2A^adult^ cells exist in the adult brain with a limited capacity for remyelination (Zhang et al., 1999). Murine oligodendroglial cells express NGFs, which are important for oligodendrocyte-neuron interactions, essential for neuronal survival, redifferentiation, and remyelination (Byravan et al., 1994). In order to study such cell-cell interactions, O-2A progenitor cells were stably transfected with the *t-neu* tyrosine kinase (Jung et al., 1995). The resulting Oli-neu cell line can be induced to differentiate *in vitro* after application of dibutyryl cAMP. In the presence of demyelinated lesions, Oli-neu cells engage with demyelinated axons but do not differentiate further to swathe the axons (Jung et al., 1995).

In the present study, expression of the myelin-related connexins Cx29, Cx32, and Cx47 (Kleopa et al., 2004; Li et al., 2004) was analyzed in differentiated and undifferentiated Oli-neu cells. Connexins are the subunits of gap junctions, which are formed by docking of two hemi-channels (connexons), each comprised of 6 connexins in adjacent cells. Today, at least 21 connexin genes are described in the murine and human genome, most of which are orthologs (Söhl and Willecke, 2003; Sonntag et al., 2009). Targeted disruption of mouse connexin genes revealed functional consequences often coinciding with pathological situations in patients suffering from mutations in the respective orthologous connexin (Willecke et al., 2002). Ablation of the connexin32 (Cx32) protein (Nelles et al., 1996) resulted in a demyelinating peripheral neuropathy (Anzini et al., 1997; Scherer et al., 1998) reverted by transgenic expression of human Cx32 in myelinating mouse Schwann cells (Scherer et al., 2005). Although abnormalities caused by Cx32 mutations found in CNS myelin are largely subtle, they fall into the category of patients suffering from the inherited peripheral neuropathy CMTX mostly caused by point mutations of the Cx32 gene (Scherer et al., 1998). Targeted deletion of the connexin47 (Cx47) gene revealed subtle vacuolization of CNS nerve fibers (Menichella et al., 2003; Odermatt et al., 2003). Cx32/Cx47-double deficient mice, however, develop a more severe CNS vacuolization coinciding with action tremor and death around 7 weeks after birth (Odermatt et al., 2003). This is reminiscent to nystagmus, progressive spasticity, and ataxia found in some patients with a mutated Cx47 gene suffering from Pelizaeus–Merzbacher-Like disease (Uhlenberg et al., 2004; Tress et al., 2011).

Connexin29 (Cx29) transcription was shown to be postnatally up-regulated in the mouse CNS concomitantly with Cx32 and Cx47 (Söhl et al., 2001a). In the CNS, Cx29 was detectable at the internodal and juxtaparanodal regions of small myelin sheaths (Altevogt et al., 2002) but did not co-localize with any of the two other oligodendroglial (Cx32 and Cx47) or the prominent astroglial connexins (Cx30 and Cx43), supposed to form an astroglial, if not panglial syncytium (Altevogt and Paul, 2004; cf. Theis et al., 2005). In the PNS, Cx29 protein was only found in the innermost layer of mouse sciatic nerve myelin (Li et al., 2002), the (juxta) paranodes, the inner mesaxon and together with Cx32 within the incisures (Altevogt et al., 2002). Cx29 hemi-channels were suggested due to their subcellular distribution in peripheral Schwann cells at the innermost layer of myelin apposing axonal Shaker-type K^+^ channels (Altevogt et al., 2002), in cochlear Schwann cells (Tang et al., 2006), and in oligodendrocytes that myelinate small caliber fibers (cf. Kleopa et al., 2010). However, transfection of Cx29 as well as its human ortholog Cx31.3 into HeLa wild-type cells neither produced significant junctional conductance nor formed functional intercellular channels or hemi-channels (Altevogt et al., 2002; Ahn et al., 2008; Sargiannidou et al., 2008). Recently, a missense mutation (E269D) in the human ortholog of Cx29 supposed to contribute to non-syndromic hearing impairment (NSHI) at least disturbed the formation of gap junctions in HeLa cell transfectants (Hong et al., 2010) although targeted deletion of the Cx29 coding region in mice does not result in any obvious phenotypical alterations or abnormalities (Altevogt and Paul, 2004; Eiberger et al., 2006).

Here we report the presence and gene structure of Cx29 in mouse sciatic nerve and brain as well as in cultured Oli-neu cells. The spliced transcript isoform contains Exon1 (442 bp), which is separated by a 4.8 kb intron from Exon2 (~3.8 kb), comprising the complete coding region. Since both the splice acceptor and the consensus motif for translational initiation partially overlap, translation efficacy is altered after splicing. Unexpectedly, out of three characterized myelin-related connexin genes Cx29, Cx32, and Cx47, only Cx29 is abundantly transcribed and translated in differentiated as well as undifferentiated Oli-neu cells. Their homologous coupling does not promote the transfer of neurobiotin, whereas immunofluorescence analyses and dye uptake measurements suggest Cx29 protein in hemi-channels. Similarly, Cx29 HeLa cell transfectants express Cx29 transcript and protein highly abundant but only show a very limited tracer coupling and dye uptake around background when compared to HeLa wild-type control.

## Materials and methods

### Sequence data acquisition and analysis

Current sequence information about the Cx29 gene is provided by the *Entrez Gene* platform of the NCBI-mediated genome browser under the GeneID: 118446. A locus tag [MGI: 2153041] of the gap junction membrane channel protein epsilon1 is provided by Mouse Genome Informatics. A genomic sequence contig [acc. no. NC_000071 or ENSMUSG00000056966] from 135358446 to 135349315 is presented which shows graphically aligned transcripts [NM_0800450] and the polypeptide sequence [Q921C1] of 258 amino acids. Furthermore, a predicted polypeptide sequence [NP_536698] of 269 amino acids is annotated to the genomic contig. This polypeptide additionally contains 11 amino acids (MLLLELPIKCR) attached to the N-terminus of Cx29 (Söhl et al., 2001a; Altevogt et al., 2002) and translation was predicted to start on exon1 supposed to be spliced to exon2 usually harboring the ATG start codon. This prediction was confirmed after applying the cross-linked *Ensembl Contig View*, the *UCSC Browser*, or the *NCBI Map Viewer*.

Two different but interrupted Cx29 cDNA clones (accession nos. AW495262 and BB644041) containing additional and overlapping sequence information at the 5′-region (5′-UTR) of the Cx29 reading frame (accession no. AJ297318) have been identified. A detailed search in the Celera's mouse genomic database [www.celera.com] yielded one contiguous Celera clone [GA_X5J8B7W67DU: 8500001::8737182], including the whole Cx29 gene. This sequence segment is quite similar to the above mentioned genomic sequence contig [acc. no. NC_000071], thus we have considered both as a common sequence segment. In order to implement an unequivocally system for position numbers of important sites (i.e., translational start site, splice donor, and acceptor sites) or primer annealing sites, we have decided to assign the A of the actual ATG start codon on exon2 with +1. Consecutive numbering further downstream will be positive in contrast to consecutive negative numbering further upstream.

Functional implications on translational efficacies of each consensus motif of translational initiation in RNA transcripts have been calculated according to the criteria of (Iida and Masuda, 1996).

### RT-PCR analysis

**Figure 1:**
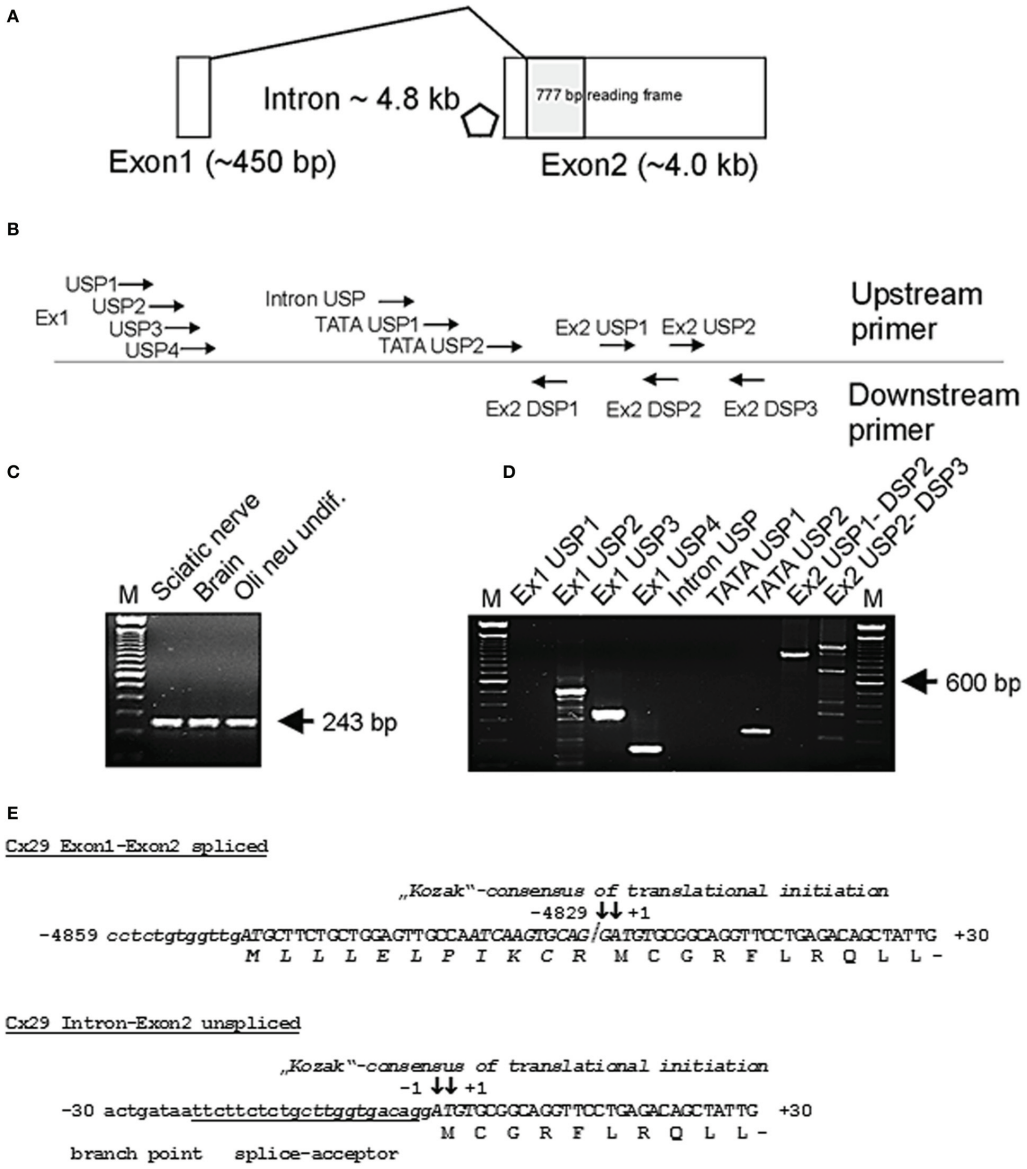
**(A)** Genomic structure of mouse connexin29 (Schematical drawing). Exons are indicated by boxes. Shaded box represents the open reading frame (ORF) of 777 base pairs (bp) encoding for 258 amino acid residues (aa). Rhombus indicates a TATA-box motif within the 4.8 kb intron (position −194 to −189). Exon1 is spliced directly to the ATG start codon. **(B)** Rough delination of primers used for subsequent RT-PCR analyses. Primer sequences, their position and orientation in the genomic sequence of Cx29 are listed in Table 1 and are accommodated to **(A)**. **(C)** RT-PCR with primers for β-actin (De Sousa et al., 1993) amplifying a single 243 bp amplicon of mouse β-actin cDNA without genomic DNA (330 bp). M = 100 bp ladder (Gibco-BRL). **(D)** RT-PCR using different primer combinations, delineated in **(B)**, and mRNA from Oli-neu cells. Seven upstream primers (Ex1 USP 1–4, Intron USP, and TATA USP 1–2) were subsequently combined with downstream primer Ex2 DSP1 to verify splicing of Exon1 to Exon2 and to roughly determine gene extension. Amplicons of 497, 284, and 127 bp (lane 2 to 4 from the left) indicate that Exon1 at least comprises 442 bp and is spliced to Exon2. The amplicon (210 bp) generated by upstream primer TATA USP2 suggests transcriptional activity downstream of a TATA-box motif (position −194 to −189). Exon2 derived primer combinations Ex2 USP1-DSP2 and Ex2 USP2-DSP3 generated fragments of 938 bp and 1195 bp extending the 3′untranslated region (3′-UTR) of Exon2 to about 3 kb. Primer DSP3 also amplified unspecific fragments under PCR conditions selected. M = 100 bp ladder (Gibco-BRL). **(E)** Partial sequences of the spliced (Exon1 and Exon2) and of the unspliced (Intron and Exon2) Cx29 transcript isoforms containing the start codon (ATG) numbered according to (+1) for the A of the ATG and (-1) for the adjacent base upstream. The coding nucleotide sequence (capital letters) is aligned to its deduced amino acid sequence. The consensus motifs of translational initiation are written in *italics*. ATG codons in use are in bold capital letters and boxed. The splice junction between exon1 and exon2 is separated by a slash. Note that a hypothetical N-terminus (gray shaded italic letters) is suggested to start at the boxed ATG on exon1. The putative branch point (boxed) and splice acceptor (underlined) overlap with the consensus initiation region of translation (Kozak, 1989; Iida and Masuda, 1996).

Reverse transcription of total RNA from mouse brain, sciatic nerve, and Oli-neu cell lines was performed according to Söhl et al. (1998). Aliquots of the transcribed cDNA [1/25 from tissue and cells (~0.1 ng)] were amplified using different combinations of Cx29 specific primers listed in Table 1 and depicted in Figure 1B. Reaction mixtures (50 μl) contained 20 mM Tris-HCl (pH 8.4), 250 μM dNTPs, 1.25 mM MgCl_2_, 50 mM KCl, 2 μM of each primer and 1 unit Taq DNA-polymerase (Promega, Madison, WI, USA). PCR was carried out for 40 cycles using a PTC-200 Thermal Cycler (MJ Research, Watertown, MA, USA) with the following program: first denaturing step at 94°C for 3 min, denaturing at 94°C for 1 min, annealing at 65°C for 1 min, elongation at 72°C for 2 min, and final elongation for 7 min. After gel electrophoresis in a 1% agarose gel Etbr stained fragments were documented (Sambrook and Russel, 2001). The integrity of all primer combinations was verified before with appropriate Cx29 genomic mouse 129/SvJ plasmid controls (Eiberger et al., 2006). Fragments of interest were excised from the gel, purified by using the QIAquick purification procedure for PCR-fragments (Qiagen, Hilden, Germany) and finally subcloned into the pGEM-T_easy_ vector system suited for cloning PCR-fragments (Promega). Fragments were commercially sequenced by AGOWA, Berlin, Germany.

**Table 1:** **Description of primers used to determine the genomic structure of mCx29**.

**Name**	**Sequence**	**Position**	**5′-linker**
Ex1 USP1	5′-gag ccg tac gag ttc tcc act gag	−5498 to −5475	–
Ex1 USP2	5′-agg cag ctc atc cct aga caa ggg	−5259 to −5236	–
Ex1 USP3	5′-act ttc cag aga tcg cgg ctt gag	−5046 to −5023	–
Ex1 USP4	5′-cca gtg tga aga ttc ctt ttg g	−4889 to −4868	*HindIII*
Intron USP	5′-act gga gct cag tgt cat gtg	−626 to −608	*KpnI*
TATA USP1	5′-aat ctg tgc tgt gct att tgg	−259 to −239	*HindIII*
TATA USP2	5′-agt aaa gat gga taa agt gtg	−157 to −137	*HindIII*
Ex2 USP1	5′-tac aaa gtt tct aag cag agg	+891 to +911	*KpnI*
Ex2 USP2	5′-gca gat ctc cca gag cac tgg	+2520 to +2540	*KpnI*
Ex2 DSP1	5′-ggt gga gtg ctg gct ctc ctg	+54 to +34	*BamHI*
Ex2 DSP2	5′-gga aag aaa aat tag aag tgg	+1829 to +1809	*BamHI*
Ex2 DSP3	5′-cac caa aca cag aca cca ttg	+3715 to +3695	*BamHI*

Position numbers refer to the genomic sequence of mCx29 available from the NCBI database available under [acc. no. NC_000071 or ENSMUSG00000056966]. Some primer additionally contain 5′-linkers with appropriate restriction sites designed according to the technical references of New England Biolabs Inc. (NEB) 2001, page 210–211.

### Northern blot analysis

Total RNA from mouse brain (C57BL/6NCrl)), from HeLa as well as Oli-neu cells was prepared with TRIzol®-reagent (GibcoBRL) according to the manufacturer. RNA (20 μg) was electrophoresed (Sambrook and Russel, 2001) and transferred to HybondN nylon membrane (Amersham International, Amersham, Bucks, UK) by capillary diffusion in 20× SSC. Northern membrane was probed by using corresponding hybridization fragments of mouse Cx29, Cx32, Cx45, and Cx47 (described in (Söhl et al., 2001a)) subsequently. Probes were ^32^P labeled, using the random primed labeling method (multiprime labelling Kit, Amersham) to a specific activity of 0.5 to 1.0 × 10^9^ cpm/μg DNA and added to fresh QuikHyb hybridization solution (Stratagene, La Jolla, CA, USA) at 1.25 × 10^6^ cpm/ml. Hybridization at high stringency was carried out for 1 h at 68°C. Filters were finally washed for 30 min in 0.1× SSC/0.1% SDS at 60°C and exposed to XAR X-ray film (Eastman Kodak, Rochester, NY, USA) with intensifying screen at −80°C. The amounts of total RNA on the Northern blot were roughly standardized by determination of the intensities of the Etbr stained 18S- and 28S rRNA. The densitometric analysis was carried out with the Biometra Scan Package (Version 4.0), Göttingen, Germany.

### Immunoblotting

Protein extracts from Oli neu cell lines and cultured HeLa cells were obtained after homogenizing by sonification in 1× Complete® protease inhibitor (Roche, Mannheim, Germany). Protein concentrations were determined after using the bicinchoninic acid protein determination kit (Sigma, Deisenhofen, Germany), according to the instructions of the manufacturer. Aliquots of 50 μg protein per lane were separated by polyacrylamide gel electrophoresis, transferred to nitrocellulose membranes (Sambrook and Russel, 2001), blocked with 1× RotiBlock® (Roth, Karlsruhe, Germany) for 1 h and incubated for 2 h at room temperature with a 1:600 dilution of polyclonal Cx29 antibodies (Zymed; Cat. No 34-4200) diluted in 1× RotiBlock. After washing (2× for 5 min and 2× for 10 min) in 1× RotiBlock immune-complexes were analyzed using ^125^I-labeled protein A.

### Immunocytochemistry

HeLa cells and Oli-neu cells were seeded on coverslips, cultivated for 3 days and washed twice in PBS for 5 min. Cells were fixed in absolute ethanol (−20°C) for 5 min, washed twice in PBS for 5 min and pre-incubated for 45 min in blocking reagent (PBS containing 4% BSA and 0.1% Triton X-100). Cryosections (12 μm) of whole C57BL/6N mouse adult brain were fixed in absolute ethanol (−20°C) for 10 min, washed twice in PBS for 5 min, and pre-incubated for 45 min in blocking reagent (PBS containing 4% BSA and 0.1% Triton X-100). For detection of Cx29, slides were incubated for 2 h with a 1:200 dilution of the affinity-purified polyclonal anti-Cx29 antibodies (Zymed; Cat. No 34-4200) at room temperature. After three washes in PBS for 5 min, respectively, tissue samples were stained for 45 min with a 1:2000 dilution of the secondary antibody Alexa (488)-conjugated goat anti-rabbit [# A11037] (MoBiTec, Göttingen, Germany). After incubation, samples were washed three times in PBS for 5 min, stained with Hoechst 33528 (Roche) and mounted in fluorescence mounting solution (DAKO, Hamburg, Germany). Fluorescent signals were recorded by using a Zeiss Axiophot photomicroscope equipped with a 63× oil immersion objective and appropriate filters.

### Plasmids

For stable transfection of HeLa cells, the translational initiation optimized Exon2 coding region of mouse Cx29 was PCR amplified from phage DNA and cloned into the expression vector pMJ green, consisting of an *Asn*I/*Stu*I fragment (2570 bp) descending from the pEGFP-N1 vector (Clontech, Palo Alto, CA, USA), which was cloned into the *Not*I/*Cla*I opened pBEHpac18 vector (~3700 bp; Horst and Hasilik, 1991) in order to construct a fusion protein of the Cx29 sequence with the enhanced green fluorescent protein at its C-terminus. The primers had a *Xho*I site at the N-terminus (primer XhoATG: 5′-CCG CTC GAG CGG CCA CCA TGT GCG GCA GGT TCC TG-3′) and a *Pst*I site behind the stop codon of the connexin gene (primer PstSTOP: 5′-CGA ATG CAT TGG CTG CAG TTC AAA ATG GCT CTT TTG C-3′) or, in case of the fusion protein, a *Pst*I-site instead of the stop codon of the Cx29 gene (primer PstGO: 5′-CGA ATG CAT TGG CTG CAG TTT AAA TGG CTC TTT TGC-3′). The PCR products were cloned with *Xho*I and *Pst*I into the pMJ green and sequenced; the resulting expression plasmids were named pCx29 and pCx29eGFP.

### HeLa cell culture and transfection

Human cervix carcinoma HeLa cells (ATCC CCL1; American Type Culture Collection, Rockville, MD, USA) were cultured in Dulbecco's modified Eagles medium (DMEM) low glucose, 2 mM glutamine, 10% fetal calf serum, 100 U/ml penicillin, and 100 μg/ml streptomycin (all from Life Technologies). HeLa transfectants were maintained in standard medium containing puromycin (0.5 mg/ml; Sigma) being cultivated in a 37°C incubator in a moist atmosphere of 10% CO_2_. HeLa cells were transfected with 20 μg of the linearized pMJ green plasmid vector containing either the pCx29 and pCx29eGFP plasmids using the calcium phosphate transfection protocol employed routinely by Elfgang et al. (1995). In brief, between 24 and 48 h after exposure to DNA, puromycin was added to the medium. Clones were picked after 3 weeks and grown under selective conditions. Alternatively, cells have been transfected using the Tfx20-reagent (Promega). Resistant clones were isolated after 2 weeks of selection with 1 μg/ml Puromycin (Sigma), and tested for Cx29EGFP expression by microscopy with an excitation wave length of 488 nm. Microinjection of dyes like Lucifer yellow, propidium iodide, EtBr, or DAPI as well as of the tracer neurobiotin was done iontophoretically in close accordance to Elfgang et al. (1995).

### Oli-neu cell culture conditions

Oli-neu cells were incubated in SATO medium containing 1% horse serum, gentamycin and 1 mM dibutyryl cAMP (dbcAMP; Sigma, Heidelberg, Germany) for differentiation during a period of at least 10–20 days at 37°C (Jung et al., 1995). Cells were plated in 35 mm Petri dishes or six-well plates on poly-L-lysine coated coverslips (Nunc, Wiesbaden, Germany), and stained for indirect immunofluorescence.

### Dye transfer measurements

HeLa or Oli-neu cells were grown on 35 mm dishes for 2–3 days. Glass micropipettes were pulled from capillary glass (World Precision Instruments, Berlin, Germany) with a horizontal pipette puller (Model P-97, Sutter Instruments, Novato, CA) and backfilled with dye solution (see below). Dyes were injected iontophoreticially (Iontophoresis Programmer model 160; World Precision Instruments) and cell-to-cell transfer was monitored using an inverted microscope (IM35; Zeiss) equipped with fluorescent illumination. Cell culture dishes were kept on a heated block at 37°C. Lucifer Yellow CH (Molecular Probes) at 4% (w/v) in 1 M LiCl was injected by applying hyperpolarizing currents for 10 s (*I* = 20 nA). Cell to cell transfer was evaluated 30 min after dye injection. Neurobiotin (N-2(2-aminoethyl)-biotinamide hydrochloride; Vector Lab, Burlingame, CA) and rhodamine 3-isothiocyanate dextrane 10S (Sigma) at concentrations of 6 and 0.4% (w/v), respectively, in 0.1 M Tris-Cl (pH 7.6) were injected by applying depolarizing currents for 10 s (*I* = 20 nA). Thirty minutes after injection, cells were washed twice with phosphate buffered saline (PBS), fixed for 10 min in 1% glutaraldehyde in PBS, washed twice with PBS, incubated in 2% Triton X-100 in PBS over night at 4°C, washed three times with PBS, incubated with horseradish peroxidase-avidinD complex (Vecor Lab) diluted 1:1000 in PBS for 90 min, washed three times with PBS, and incubated in 0.05% diaminobenzidine, 0.003% hydrogen peroxide solution for 30 s. The staining reaction was stopped by washing three times with PBS. Cell-to-cell transfer was quantified by counting the number of stained cells neighboring the microinjected cell.

### Dye uptake in oli-neu cells

Stock solutions of fluorescence dyes were prepared in 150 mM LiCl [110 μM Lucifer Yellow CH (LY)] or in PBS (5 mM or 12.5 mM Etbr). For visualization of dye uptake, 0.5 μl of stock solutions were applied at room temperature through a glass micropipette over the area of the culture plate to be evaluated, and 2 min later, the dye was washed away and replaced with recording medium (SATO). Alternatively, 2.5 μl Etbr (1 mg/ml) was added directly to a 6 cm culture dish, incubated for 4 min, washed and subsequently replaced by fresh medium. The former described local application of the dye, however, produced only little background and was thus preferred. The gap junction blocker heptanol and octanol have been applied for 5 min, 1 mM just before dye application. In permeabilized cells, Etbr labelling was detectable as fluorescence of the nuclei. The retained dye was monitored using an inverted microscope (IM35; Zeiss) equipped with fluorescent illumination and documented by a Powershot G50 digital camera (Canon). For time-lapse recordings of dye uptake, the fluorescence exposure times were usually 5–10 s, with 15 s between each fluorescence image. This procedure was already and successfully applied to cultured astrocytes (Contreras et al., 2002) and thus adapted for Oli-neu cells.

## Results

### Genomic structure of mouse connexin29

Untranslated sequence information (5′-UTR) of two interrupted Cx29 cDNA clones have been aligned in the genomic Cx29 sequence from position −5049 to −4829 (see sequence data acquisition and analysis), representing a putative untranslated Exon1 of 220 bp with a splice-donor site (CAG↓**GT**AAAT) at its 3′-end, that contains all criteria of canonical splice-donor sites (Padgett et al., 1986). It is separated by an intron of about 4.8 kb from the coding region (shaded box; Figure 1A). RT-PCR primer sequences and template positions to verify functional splicing of Cx29 Exon1 to Exon2 are listed in Table 1 and schematically delineated in Figure 1B.

Quality of cDNA pools generated from total RNA of mouse sciatic nerve, brain and Oli-neu cells have been tested using a specific primer combination to amplify β-actin (De Sousa et al., 1993). No residual genomic DNA contamination was detectable (330 bp) whereas the 243 bp cDNA β-actin amplicon implied proper cDNA synthesis (Figure 1C).

A subset of upstream primers covering Exon1 (220 bp) and the genomic region further upstream, was combined with intron-spanning Exon2-specific downstream primer Ex2 DSP1 (see Table 1 and Figure 1B) to produce a distinct pattern of amplicons, enabling a rough estimation of the putative Exon 1 size (Figure 1D). The shortest amplicon of 127 bp (lane 4) was subcloned, sequenced, and confirmed the anticipated splice pattern deduced from both cDNA clones. Thus, Exon1 of Cx29 is functionally expressed and spliced to Exon2 in at least mouse sciatic nerve, mouse brain as well as in Oli-neu cells (undifferentiated) (Figure 1D). Larger amplicons of about 284 and 497 bp (lane 2 and 3; Figure 1D) let extend Exon1 to at least 442 bp (see also Figure 1A).

A further objective was to determine the approx. 5′-extention of Cx29 Exon2, since the corresponding splice acceptor site and the consensus motif of translational initiation (Kozak, 1989) nearly coincide in a sequence of 12 bp (depicted *in italics* in Figure 1E).

Therefore, three upstream primers (Table 1) should anneal between Exon 1 and 2 (two of them enframe a putative TATA-box motif), which were combined with downstream primer Ex2 DSP1 (see Figure 1B and Table 1). Unexpectedly, the intron-derived primer (TATA USP2) downstream of the TATA-box motif (rhombus; Figure 1A) was able to generate an amplicon of 210 bp, suggesting the initiation of transcription. Cloning and sequencing identified the genomic sequence containing the native splice acceptor site (Figure 1E). Thus, an additional unspliced transcript isoform might be expressed from the Cx29 gene. The fact that the consensus motif of the splice acceptor site is in such a proximity to the ATG start codon leaves only the base guanine in front of the ATG unchanged after splicing, which, however, does not severely decrease (only 12%) the efficacy of translational initiation compared to the unspliced Cx29 transcript isoform (Figure 1E, Table 2). An alternative ATG in frame within exon1, suspected to initiate translation of an elongated Cx29 protein with additional 11 N-terminal amino acid residues [NP_536698] is covered by a consensus motif of a very low translational efficacy (1.7) inappropriate to initiate translation (Iida and Masuda, 1996; Kozak, 1997). Instead, translation (efficacy 3.3) is more likely to start with the ATG located further upstream (Table 2) but being out of frame. Conclusively, these data confirm that either the spliced or the unspliced consensus motif around the ATG at position (+1) will sustainably initiate translation of the Cx29 protein (Figure 1E).

**Table 2:** **An evaluation matrix suggested by Iida and Masuda (1996) helps to calculate the hypothetical efficacy of suspected translational initiation sites, termed more generally consensus motifs for translational initiation**.

**Consensus motif of translation**	**Sequence**	**Position**	**Efficacy**	**% efficacy**
High impact motif	[ccaggggacaacATGg]	–	6.7	100
Cx29 unspliced motif	[cttggtgacaggATGt]	−12 to +4	5.0	75
Cx29 spliced motif (Ex1 to Ex2)	[atcaagtgcaggATGt]	−4839 to +4	4.2	63
Cx29 exon1 motif (out of frame)	[agcctgccaggaATGa]	−4975 to −4960	3.8	57
Cx29 exon1 motif (in frame) see Figure 1E	[cctctgtggttgATGc]	−4850 to −4835	1.7	27
Cx29 intron motif	[ctttgcagacccATGg]	– 102 to −87	3.3	50
Most inappropriate motif	[tggattaggtgtATGc]	–	0.3	0

Sixteen bases around the bold ATG of the genomic sequence of connexin29 [acc. no. NC_000071 or ENSMUSG00000056966] are included, each. Both consensus motifs, which confer the highest (6.7) and the lowest (0.3) absolute efficacy in terms of translational initiation are included in order to determine relative (%) efficacies. Note the relatively low efficacy (1.7) of the consensus motif around the in-frame ATG of exon1 which is suggested to initiate translation of a connexin29 protein harboring eleven additional amino acid residues at its N-terminus (MLLLELPIKCR).

Two independent primer combinations located in the putative 3′-untranslated region (3′-UTR) of exon2 should determine the downstream extension of the Cx29 transcript. PCR-fragments of calculated sizes (Figure 1D) indicated that about 3 kb might be considered as the (3′-UTR) of Exon2. Therefore, a predicted tandem termination region containing totally four polyA-signals (AAUAAA) from position +4283 to +4415 is likely to be used (Genescan tool/HUSAR/Heidelberg). Finally, one can estimate the size of both Cx29 transcript isoforms. Each contains an exon2 of about 4.3 kb, but one additionally obtains a spliced exon1 of 440 bp, and the other merely will contain an upstream extension of 160 bp.

### Northern and immunoblot hybridization

**Figure 2:**
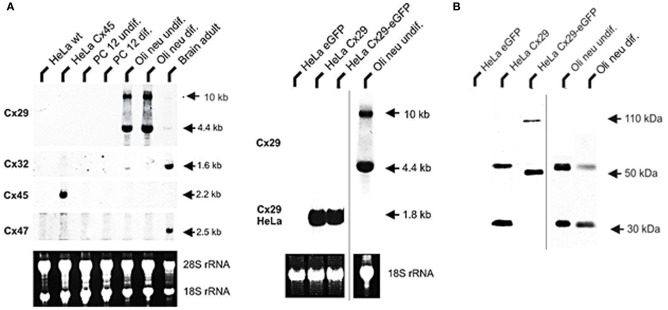
**Northern blot analysis of total RNA from HeLa, PC12, Oli-neu cells, and mouse adult brain. (A)** A 4.4 kb signal representing Cx29 expression was detected highly abundant in undifferentiated as well as differentiated Oli-neu cells and weakly in brain. A 1.6 kb signal of Cx32 expression is distinctly visible in RNA from adult brain (100%) as control but is hardly detectable (3 vs. 14%) in Oli-neu cells (diff. and undif.), respectively. Cx47 expression (2.5 kb) is also evident in adult brain but missing in Oli-neu cells. No signals of oligodendroglial connexins were seen in HeLa wild type (wt), HeLa Cx45 transfectants, and PC12 cells (diff. or undif.). Additional hybridization signals at about 10 kb are visible after hybridizing the Cx29 probe against RNA of Oli-neu cells as well as from adult brain. HeLa cells stably transfected with the tandem cloned connexin29 and eGFP reading frames (both of ~800 bp) either separated by a stop-codon or directly fused, yielded hybridization signals of the expected 1.8 kb. The blot was standardized by measuring the intensities of both the Etbr stained 18S- and 28S-rRNA. All signals have been documented after 3 weeks of exposure. **(B)** Immonoblot analysis of Cx29 and Cx29-eGFP stably transfected HeLa cells and Oli-neu cells (dif. and undif.) using the Cx29 polyclonal antibodies (Zymed). Two signals of about 30 and 56 kDa were prominent in Cx29 HeLa cells and Oli-neu cells. In the Cx29-eGFP HeLa cells, however, both signals seemed to be shifted to about 50 and 110 kDa due to the appended eGFP coding region.

Northern blot hybridizations using probes against Cx29, −32, −45, and −47 underscored only an abundant Cx29 expression in both types of differentiated as well as undifferentiated Oli-neu cells. Interestingly, expression of Cx47 is not present and Cx32 was only found weakly in undifferentiated Oli-neu cells (Figure 2A). This was, however, unexpected since Cx32 and Cx47 are connexin genes characterized to occur in oligodendrocytes (Kleopa et al., 2004; Li et al., 2004).

The absence of any signal in the lanes of the established rat adrenal pheochromocytoma cell line (PC12), having a neuronal background (Greene and Tischler, 1976) and used as control, underlined that glial connexins are absent from this cell type. RNA from HeLa wild-type and HeLa Cx45 transfectants was additionally blotted in order to exclude possible cross reactions of the Cx47 hybridization probe due to high sequence similarities with connexins45 (Teubner et al., 2001). As expected, hybridization probe against Cx45 only gave a signal with Cx45 transfected HeLa cells, coinciding well to the current opinion that Cx45 is expressed in neurons (Maxeiner et al., 2003) but not in PC12 cells. Concerning Cx29, however, two hybridization signals (4.4 kb and ~10 kb) have been detected. Whereas the shorter fragment could be readily explained by summing up both exons amplified by RT-PCR (~4.6 kb), the larger signal is likely to represent the unspliced heteronuclear RNA of the Cx29 transcript, containing still the 4.8 kb intron.

Immunoblotting of lysates harvested from stably transfected Cx29 HeLa cells and from undifferentiated Oli-neu cells identified two different protein fractions of about 30 and 56 kDa (Figure 2B). This pattern is quite similar to results obtained after using homogenates from mouse sciatic nerves and lysates of transiently transfected Cx29 HeLa cells (Li et al., 2002), except that a third immunosignal of about 70 kDa was not detectable in our blots. Stably transfected HeLa cells expressing a fusion protein of Cx29 and eGFP instead showed a shift in both signals to 50 and 110 kDa. Thus, both HeLa cell lines express highly abundantly either the proper Cx29 protein or the fusion protein consisting of Cx29 and eGFP.

### Cx29 immunofluorescence analyses of stably transfected HeLa cells

HeLa cells have already been transiently transfected with Cx29 cDNAs in order to establish cultured cells and protein lysates serving for positive controls to test the manufactured polyclonal antibodies against Cx29 (Zymed). Immunosignals have been detected in the cytoplasm and the periphery of these transfectants. However, no functional dye or tracer transfer studies have been undertaken then (Li et al., 2002).

**Figure 3:**
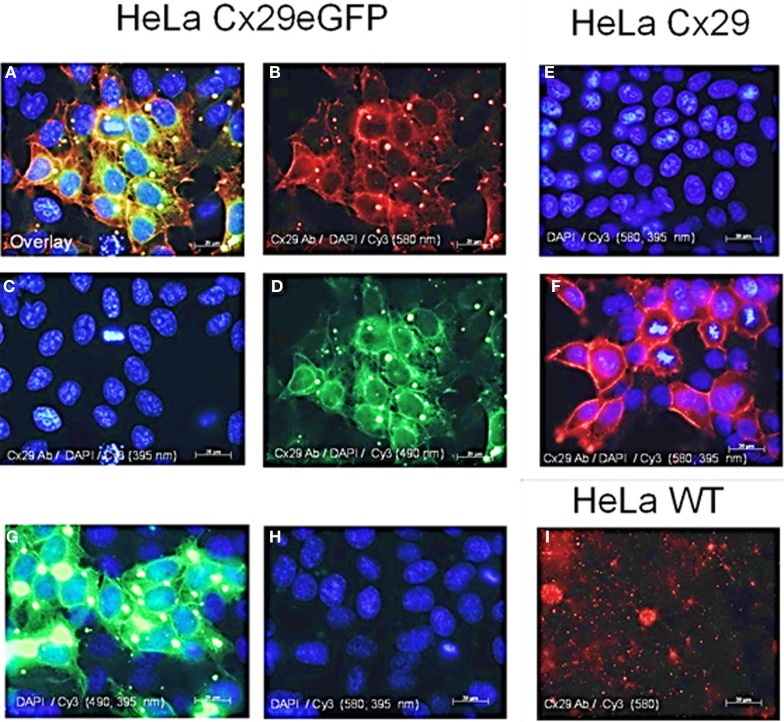
**Immunofluorescence analysis of stably transfected Cx29-HeLa cells. (A–D)** HeLa cells transfected with a construct coding for Cx29-eGFP fusion protein. **(A)** Merged picture of **(B–D)**. **(B)** Immunofluorescence after applying the Cy3 filter (580 nm) indicating Cx29 antibody distribution. **(C)** Staining of the cellular nuclei of the Cx29-eGFP transfectants with DAPI. **(D)** Green fluorescence of the eGFP reflects the distribution of the Cx29-eGFP fusion protein. **(E)** Staining of the cellular nuclei of the Cx29 transfectants with DAPI. Application of single secondary Cy3 antibody excludes cross-reactivity of the secondary antibody. **(F)** Distribution of the Cx29 protein and cellular nuclei after a double staining with Cx29 antibodies, DAPI, and Cy3. **(G,H)** Cy3 and filter control. Omitting the primary Cx29 antibody but instead applying DAPI and Cy3 allows to control if the intense green signals detectable through the FITC filter (490 nm) in **(G)** also pass, at least in traces, through the Cy3 filter (580 nm), thus mimicking Cx29 signals. Furthermore, the Cy3 secondary antibody does not cross react. **(I)** No cross reactivity is seen with HeLa wild type cells, neither with Cx29 antibodies nor with secondary Cy3 antibodies.

Distribution of Cx29 protein in stably transfected Cx29- and Cx29-eGFP cells is shown in Figure 3. The Cx29 protein is transferred to the membrane of the transfected HeLa cells forming gap junction like plaques between neighboring cells (Figure 3F). The Cx29-eGFP fusion protein, however, is only partially transported into the plasma membrane while aggregates also remained within the cytoplasm (see Figures 3A,B,D) indicated by the red immunofluorescence (Figure 3B) of Cx29 protein, that completely overlap with the green fluorescence evoked by the eGFP protein after merging both fluorescence signals (Figure 3A). Here, gap junction plaques seem less abundant compared to the Cx29 transfected HeLa cells, omitting eGFP. These results indicate that both transfected HeLa cell lines (Cx29 and Cx29-eGFP) express and process Cx29 protein to their membranes so that gap junction like plaques can be formed between adjacent cells. It cannot be excluded that the observed aggregation of the Cx29-eGFP fusion protein might be induced by the eGFP protein. Therefore, Cx29-eGFP HeLa transfectants were omitted from further functional studies.

### Dye and tracer injections into Cx29 HeLa cells

**Figure 4:**
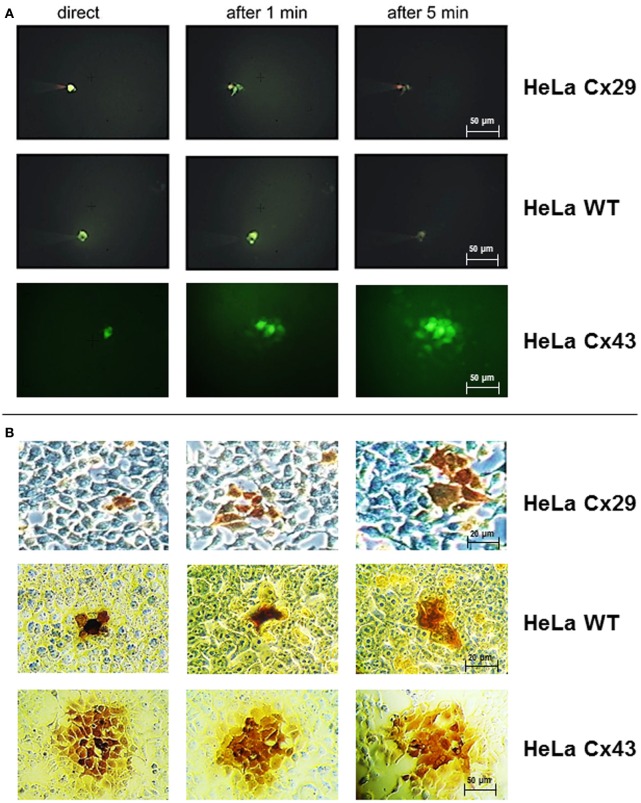
**Dye and tracer injections into Cx29-transfected HeLa cells, untransfected HeLa wild-type cells, and Cx43-transfected HeLa cells. (A)** Absence of any spreading of microinjected Lucifer Yellow into neighboring cells in both HeLa wild type as well as Cx29-transfected cells indicated the impermeability of Cx29 gap junction channels for this dye. In contrast, Lucifer Yellow spreads readily into neighboring Cx43-transfected HeLa cells after injection. **(B)** Corresponding three single neurobiotin microinjections in Cx29-tranfected, wild-type, and Cx43-transfected HeLa cells fixed and stained after 30 min. The transfer of neurobiotin into an average of 2.4 of about 7 next neighboring cells is similar after injection of HeLa wild-type and Cx29-transfected HeLa cells and in contrast to Cx43-transfected HeLa cells. In these cells tracer commonly spreads into the third order of neighboring cells.

To examine if molecules can pass through Cx29-mediated gap junction channels, permeability was investigated after injection of Lucifer Yellow (M_r_ 488, net charge −2) or neurobiotin (M_r_ 287, net charge +1) into one cell of a cluster. Neither HeLa wild-type cells nor Cx29 stably-transfected HeLa cells showed dye transfer after injection with Lucifer Yellow (Figure 4A). Cx43 stably-transfected HeLa cells used as control demonstrated the frequently described abundant spread of Lucifer Yellow (around 80% of first order neighboring cells surrounding the injected one; *n* = 15; see Elfgang et al., 1995), that have been measured after 5 min.

The transfer of the tracer molecule neurobiotin was examined up to 30 min after injection. In both HeLa wild type cells (*n* = 27) and Cx29 stably-transfected HeLa cells (*n* = 15) only about 35% of the first order neighboring cells were stained (Figure 4B), probably due to a background spread after the long incubation time after injection. Additionally, tracer transfer to higher order surrounding cells was always negligible and shorter incubation times as well as changing to Etbr or Propidium iodide (data not shown) revealed no difference between stably-transfected and wild-type cells. As control, transfer between Cx43 stably-transfected HeLa cells (*n* = 15) was about 82% of first order neighboring cells, 99 and 53% of second, respectively third order neighboring cells after 5 min. Thus, there is no coupling measurable between Cx29 stably-transfected HeLa cells.

### Cx29 immunofluorescence analyses of oli-neu cells

**Figure 5:**
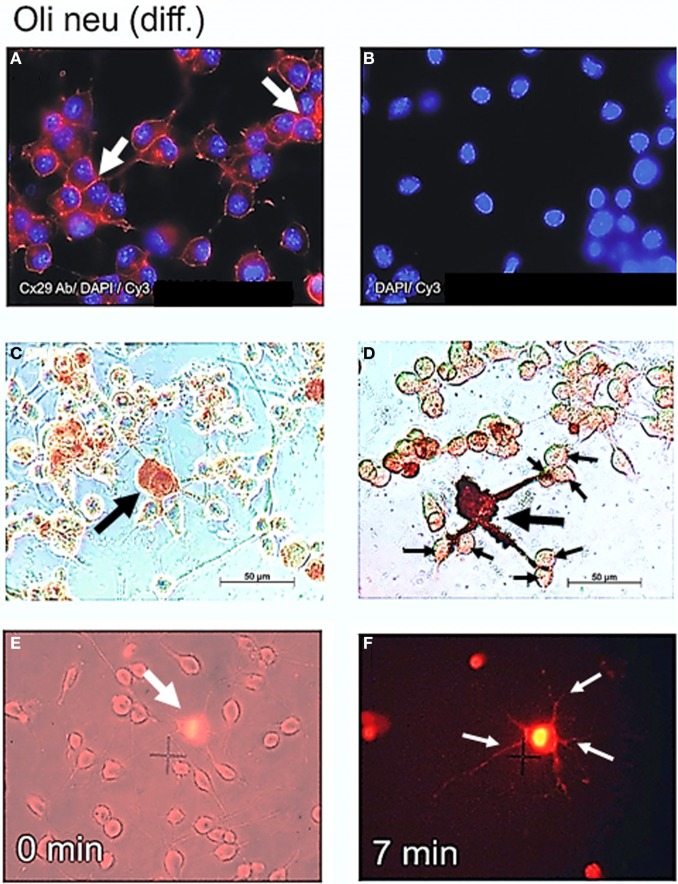
**Cx29 immunofluorescence analyses and microinjections of differentiated Oli-neu cells. (A)** Distribution of the Cx29 protein mostly in plasma membranes (arrows) applying Cx29-specific and Cy3 coupled secondary antibodies in relation to the cellular nuclei after DAPI staining. **(B)** Omitting primary Cx29 antibodies excluded cross reactivity of Cy3 antibodies with Oli-neu cells. (**C** and **D**) Two examples of Oli-neu cells microinjected with neurobiotin and stained thereafter (arrow) **(C)** No neurobiotin transfer into both cells directly attached below was detectable. **(D)** After filling one cell (large arrow), neurobiotin readily spreads into the three protrusions but did not migrate further into adjacent cells at their ends (small arrows). **(E)** Combined UV light- and trans-illumination identified the Etbr-microinjected Oli-neu cell (arrow). **(F)** Only the Etbr filled soma and the protrusions of the injected cell are faintly visible after 7 min (arrows).

Northern blot as well as immunoblot results suggested that Cx29 is highly expressed in both undifferentiated as well as in differentiated Oli-neu cells. In order to determine the subcellular distribution of Cx29 protein, immunofluorescence analyses have been performed with differentiated Oli-neu cells (and as a completion with undifferentiated Oli-neu cells, see Figure A2). Immunofluorescence signals of antibodies against Cx29 (Figure 5A) implied that the Cx29 protein is transported properly into their plasma membranes, regardless if cells are isolated or clustered in small groups. Omitting the primary antibody leads to an absence of any immunofluorescence signal, excluding cross reactivity of the secondary antibody Cy3 (Figure 5B). Furthermore, due to stronger signals between closely attached cells, it became apparent, that there might be an accumulation of Cx29 resulting upon formation of gap junction plaques between adjacent cells. Thus, under cell culture conditions differentiated Oli-neu cells might constitute a cell line to study functional Cx29 gap junction coupling. Long-term culture procedures will hence support the attachment of growing cells, whereas culturing for a shorter period might allow examination of hemi-channel activity within separated cells.

### Measurement of direct gap junction coupling between oli-neu cells

Highly abundant subcellular distribution of Cx29 protein in the plasma membranes of differentiated Oli-neu cells (see Figure 5A) suggested direct coupling between closely attached cell bodies or their developed protrusions. However, after microinjection of Lucifer yellow and neurobiotin into differentiated Oli-neu cells, either adjacent to neighboring cells (Figure 5C) or linked by protrusions to its neighbors (Figure 5D) these tracers were kept within the injected cells and did never spread into attached or connected cells. Figure 5D underlines that neurobiotin is only accumulated within the injected cell and its protrusions. These results could be confirmed after microinjection of Etbr into differentiated Oli-neu cells (Figures 5E,F). From totally 20 Etbr injections only two exceptions of Etbr spread into the next neighbor cells was found. Microinjected cells had an average of 0.7 next neighbors and about 6 cells to which protrusions are forwarded.

### Dye uptake measurements of oli-neu cells

**Figure 6:**
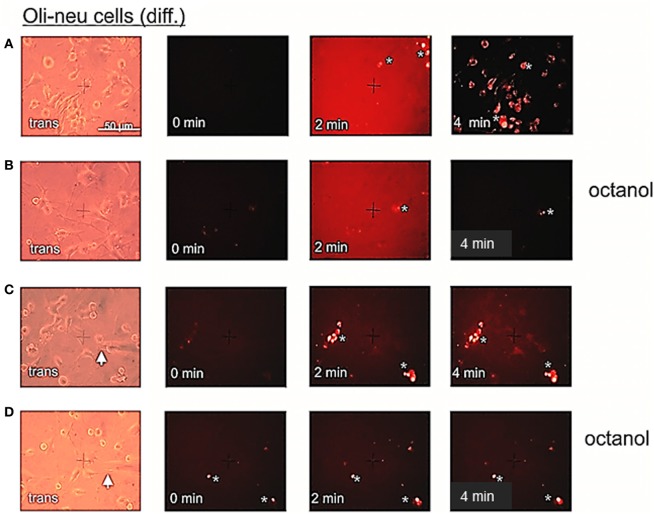
**Etbr uptake by differentiated Oli-neu cells can be blocked by octanol. (A)** Phase contrast image (trans) of a culture dish sector before application of 2.5 μl Etbr (1 mg/ml) directly to the medium (0 min). After 4 min, cells were briefly washed in PBS and photographed thereafter. Oli-neu cells of a different sector are slightly stained with Etbr. **(B)** Cells have been incubated with 1 mM octanol 5 min before dye application (0 min). After 4 min, cells have been washed and the same sector has been photographed. **(C)** Direct application (0 min) of 0.5 μl Etbr (5 mM) by a micropipette (white arrow) to the cultured cells. After 4 min, a faint Etbr staining of all cells next to the pipette tip is visible. **(D)** Cells have been incubated with 1 mM octanol 5 min before dye application. After 4 min very faint Etbr staining of cells in the vicinity of the pipette tip was detectable. Presumably cellular artifacts are directly stained after application of Etbr in **(A,B,C)** and **(D)**.

The high abundance of Cx29 protein in the plasma membranes of differentiated Oli-neu cells might implicate the formation of hemi-channels. In order to functionally determine detached hemi-channels, we applied Etbr to differentiated Oli-neu cells in culture and also tried to inhibit putative dye uptake by applying commonly used gap junction blockers like heptanol and octanol. This procedure was established by Contreras et al. (2002) with cultured cortical astrocytes. The authors demonstrated that an induced opening of distant connexin43 gap junction hemi-channels by inhibition of glycolytic and oxidative metabolism could be blocked significantly i.e., by octanol. In our study, we omitted metabolic inhibition of the cultured Oli-neu cells in order to initially analyze Cx29 hemi-channel function under normal physiological conditions. In 12 independent applications Etbr was directly added to the culture media on the top of the cells in the visible field and an average of 4.9 presumably cellular artifacts showed an immediate dye uptake after ~55 s, whereas an average of 20.5 cells in the visual field weakly took up the dye just after 2 min. To reduce background staining of Etbr in culture media, cells have been washed and documented after 4 min (Figure 6A). Application of octanol to the cell culture 5 min before dye application (*n* = 13) completely blocked the uptake of Etbr in about 19.9 cells of the visual field, whereas the 4.8 presumably cellular artifacts showed staining already after ~30 s (Figure 6B). To increase the sensitivity of the uptake measurement the concentration of the applied Etbr was five-fold decreased. Again, Etbr uptake (*n* = 6) without gap junction blocker was seen on average in 7.6 presumably cellular artifacts after ~20 s and in all 22.8 cells of the field after ~4 min (Figure 6C). Application of octanol before the release of Etbr (*n* = 2) again completely blocked the uptake of dye in all 16 cells whereas 5 cellular artifacts on average readily took up the dye after 15 s (Figure 6D). We additionally applied the gap junction blocker heptanol in a serial trial of four micropipette applications of Etbr at a higher concentration (12.5 mM). In no case dye uptake could be monitored in at least 20 visible cells but the mean 2 putative cellular artifacts had already incorporated the dye after 3.6 s (not shown). The results of the dye uptake measurements can be summarized accordingly: (1) Although Cx29 protein is prominent in the plasma membrane of single Oli-neu cells, the uptake of the extracellular dye Etbr remains dispensable. (2) The subtle uptake of the dye after, at least 4 min could clearly be prevented by gap junction hemi-channel blockers like octanol or heptanol.

It is now tempting to speculate, whether putative hemi-channels are largely closed in the plasma membrane of Oli-neu cells under normal culture conditions. A presumed transfer through these hemi-channels (Li et al., 1996) would allow a subtle uptake of Etbr that could be prevented after application of commonly used gap junction blockers. Already before, there have been no indications of functional hemi-channels of mouse Cx29 (Altevogt et al., 2002) and its human ortholog Cx31.3 (Sargiannidou et al., 2008) in transfected HeLa cells.

## Discussion

### Gene structure of mouse connexin29

A close genomic characterization implied that a spliced as well as unspliced transcript isoform can be expressed from mouse Cx29 locus. Because both the splice acceptor and consensus motif for translational initiation nearly overlap, these isoforms indeed differ in their respective motifs but only with negligible effect on translational efficacy (Iida and Masuda, 1996). Northern blot hybridization revealed a prominent 4.4 and 10 kb band comprising the expression of both transcript isoforms that coincides in sciatic nerve and brain as well as in Oli-neu cells. This is reminiscent of the expression of different Cx32 transcript isoforms in sciatic nerve (Neuhaus et al., 1996; Söhl et al., 2001b).

Calculation of translational efficacies of various consensus motifs let it appear implausible that the prominent but predicted ATG start codon on exon1 [NP_536698], being in frame with the coding region on exon2 after splicing, is used *in vivo*. However, if cell-type specific factors might support initiation of translation at least partially at this ATG on exon1, then Cx29 proteins are likely to contain additional N-terminal amino acids (MLLLELPIKCR) unusual to other connexin proteins.

### Expression of mouse connexin29

**Figure A1:**
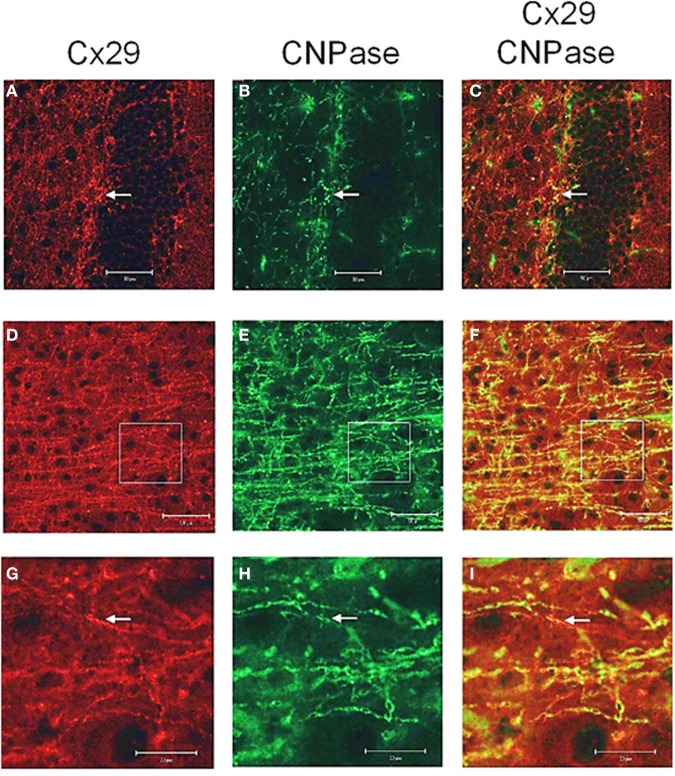
**Laser scan microscopy immunofluorescence analyses of Cx29 protein expression in the hippocampus (A–C) and in the neocortex (D–I) of the adult mouse brain.** Pictures **(G–I)** represent enlargements of the white squares present in pictures **(D–F)**, respectively. Cx29 immunofluorescence signals **(A,D,G)** co-localize with myelin-associated CNPase (2′, 3′-cyclic nucleotide 3′-phosphodiesterase). CNPase is present in the plasma membrane of oligodendrocytes and their processes, in periaxonal membranes and the inner mesaxons, the outer processes, the paranodal myelin loops, and the “incisure-like” membranes **(B,E,H)**. Merged pictures are **(C,F,I)**. Arrows in **(A,B,C)** indicate co-localization of Cx29 and CNPase in the soma of an oligodendrocyte. Arrows in **(G,H,I)** point to a co-localization of Cx29 and CNPase in myelinated fibers. However, Cx29 is hardly co-localized but present in the same neural structures as the myelin associated CNPase. This expression profile strengthened the credibility of the used Cx29 antibodies (Zymed), which was tested on tissue sections of Cx29 (–/–) deficient mice (Eiberger et al., 2006).

**Figure A2:**
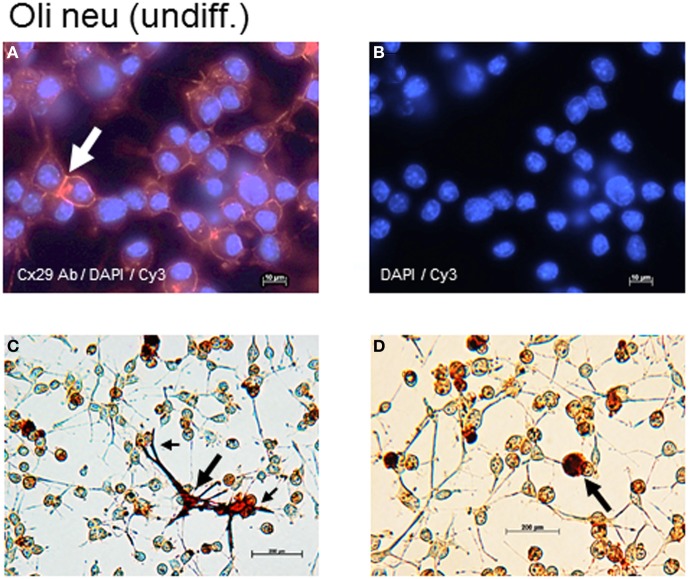
**Cx29 immunofluorescence analyses and microinjections of undifferentiated Oli-neu cells. (A)** Distribution of the Cx29 protein mostly in plasma membranes (arrow) applying Cx29-specific and Cy3 coupled secondary antibodies in relation to the cellular nuclei after DAPI staining. **(B)** Omitting primary Cx29 antibodies excluded cross reactivity of Cy3 antibodies with Oli-neu cells. **(C,D)** Two examples of Oli-neu cells microinjected with neurobiotin and stained thereafter (arrow). **(C)** After filling one cell (large arrow), neurobiotin readily spreads into the various protrusions but did not pour into the attached cells at the ends (small arrows). **(D)** No neurobiotin transfer into the next neighboring could be demonstrated.

Zymed anti-Cx29 antisera gave similar and intense results when applied to Oli-neu cells in culture or to Oli-neu lysates in immunoblots, respectively. Mouse brain regions enriched of myelinated oligodendrocytes also showed intense immunofluorescent signals after antibody application (see Figure A1). Stable Cx29-transfected HeLa cells yielded a punctuated staining pattern in their membranes. Immunoblotting of lysates from stable Cx29-transfected HeLa cells also detected two different protein fractions of about 30 and 56 kDa, comparable with lysates of either Oli-neu cells or tissue homogenates collected from sciatic nerve (Li et al., 2002). The specificity of the applied Cx29-antibodies was finally proven after targeted deletion of the Cx29 gene from the mouse genome. This also unraveled the fact that the 56 kDa protein fraction must be related to Cx29, possibly a stable dimer form of Cx29, since it disappeared like the 30 kDa signal after immunoblotting of brain and sciatic nerve homogenates from Cx29 (–/–) mice (Eiberger et al., 2006). Even the intense (often cytoplasmic) immunofluorescence labeling within presumably oligodendroglial cells of the CNS from wild-type animals was absent in Cx29 deficient mice (Eiberger et al., 2006). It became evident that these cytoplasmic signals do not reflect an unspecific cross-reactivity of the antibody to myelin, but represent Cx29 protein processed or stored within oligodendroglial cells. Therefore, the strong immunofluorescence signals in Oli-neu cell and the immunoblot signals of their lysates might reflect high abundant Cx29 protein expression. Conclusively, the Oli-neu cell culture system contains a high abundance of Cx29 mRNA and protein, thus facilitating the analysis of transcript structures as well as protein localization.

### Localization and function of mouse connexin29

In contrast to Cx32 and Cx47, only little is known about the precise position and function of Cx29 protein within myelin. Cx29 seems to be located at the internodal and juxtaparanodal regions of small myelin sheaths (Altevogt et al., 2002) but does not co-localize with the two other oligodendroglial connexins (Altevogt and Paul, 2004). Homotypic Cx29 channels within intracellular membranes of myelin or hemi-channels are suspected to allow glial uptake of K^+^ from the small, extracellular space between axon and Schwann cells (Brophy, 2001) or likewise between axon and oligodendrocyte (Altevogt and Paul, 2004). With respect to their immunolocalization, Cx29 (hemi)-channels might exist in close vicinity to Kv 1.1 and Kv 1.2 potassium channels, also predicted in juxtaparanodal axonal membranes (Arroyo and Scherer, 2000; Altevogt et al., 2002).

However, disruption of the Cx29 gene leads to no obvious phenotypical alteration or abnormalities. Cx29 (–/–) mice are viable, healthy, and fertile (Altevogt and Paul, 2004; Eiberger et al., 2006). Both studies speculated that the function of Cx29 is either dispensable or compensated by redundancy of both the other oligodendroglial connexins Cx32 and Cx47, but the sub-cellular detachment of Cx29 to Cx32 and Cx47 is obscuring this hypothesis. Nevertheless, replacement of the Cx29 coding region by a LacZ reporter gene identified novel Cx29 expressing cell types and tissues, like Bergmann glia cell of the cerebellum (Altevogt and Paul, 2004) or the postnatal cochlea (Eiberger et al., 2006). Interestingly, at least one missense mutation (E269D) in the human ortholog of Cx29 is discussed to contribute to NSHI. This mutation disturbs, at least, the formation of gap junctions after co-transfections in the HeLa cell culture system (Hong et al., 2010).

Here we have expanded the unexpected expression profile of Cx29 on Oli-neu cells, which derived from stably *t-neu* tyrosine kinase transfected O-2A cells that provide a developmental state comparable to neonatal oligodendroglial precurser cells *in vivo* (Jung et al., 1995). This extended our Northern blot result with RNA from different perinatal stages of mouse total brain, implying that Cx29 is not up-regulated before postnatal day 7 (Söhl et al., 2001a). Thus, it might be possible that high abundance of Cx29 expression reflects an auspicious side effect due to transfection of the *t-neu* tyrosine kinase. In this case, differentiated Oli-neu cells might serve as a suitable cell culture system to study the function of Cx29, since both the other oligodendroglial connexins Cx32 and Cx47, are rather absent from these cells. Interestingly, induction of differentiation with dibutyryl cAMP continuously suppressed Cx32 and Cx47. This is in contrast to the co-cultivation of Oli-neu cells with non-touching astrocytes, which have a significant impact on the expression levels of genes supporting myelination. Both Cx29 and Cx47 have been among these genes, being up-regulated due to the proximity of astrocytes (Iacobas and Iacobas, 2010).

Cx29 gap junction channels remain closed between neighboring Cx29, Cx29-eGFP HeLa cell transfectants, between undifferentiated and differentiated Oli-neu and their protrusions, respectively. This outcome is in accordance to other studies (Altevogt et al., 2002; Ahn et al., 2008). Although the oligodendroglial precursor cell line Oli-neu has, compared to stably-transfected HeLa cells, advantages like proper cytoplasmic conditions enriched with putative co-factors for channel function or the native genomic vicinity around the Cx29 gene, it rather has no impact on Cx29 channel opening. Despite a more convenient environment, selective signals for opening still seem to be needed, yet. Again, proximity of astrocytes (Iacobas and Iacobas, 2010) might support Oli-neu cells with distinct signals for channel opening. Co-cultures of Oli-neu cells with neurons *in vitro* and *in vivo* are likely to produce also important extracellular signals.

In accordance to the localization of Cx29 protein in the plasma membrane of single Oli-neu cells, we cannot exclude Cx29 to form hemi-channels. However, dye-uptake studies after application of two different hemi-channel blockers suggested that putative hemi-channels are largely closed under recommended cell culture conditions, which might diverge from physiological condition *in vivo*. Even though extracellular calcium (Hofer and Dermietzel, 1998) or metabolic stress (Contreras et al., 2002) have been reported to induce Cx43 hemi-channel opening in astrocytes, the physiological signals to open Cx29 hemi-channels under non stressed conditions remain to be discovered. Therefore, this Oli-neu cell culture system might represent a well suited tool to functionally explore these signals.

### Conflict of interest statement

The authors declare that the research was conducted in the absence of any commercial or financial relationships that could be construed as a potential conflict of interest.

## Abbreviations

RT-PCR: polymerase chain reaction after reverse transcription
Cx: connexin
UTR: untranslated region
CNS: central nervous system
PNS: peripheral nervous system
Etbr: ethidiumbromide
eGFP: enhanced green fluorescence protein
NGF: nerve growth factor
CMTX: Charcot-Marie-Tooth disease X-chromosomal.
